# Genome-Wide Evolution and Comparative Analysis of Superoxide Dismutase Gene Family in Cucurbitaceae and Expression Analysis of *Lagenaria siceraria* Under Multiple Abiotic Stresses

**DOI:** 10.3389/fgene.2021.784878

**Published:** 2022-02-08

**Authors:** Shamsur Rehman, Arif Rashid, Muhammad Aamir Manzoor, Lingling Li, Weibo Sun, Muhammad Waheed Riaz, Dawei Li, Qiang Zhuge

**Affiliations:** ^1^ Co-Innovation Center for Sustainable Forestry in Southern China, Key Laboratory of Forest Genetics and Biotechnology, College of Biology and the Environment, Nanjing Forestry University, Ministry of Education, Nanjing, China; ^2^ State Key Laboratory of Tea Plant Biology and Utilization, Anhui Agricultural University, Hefei, China; ^3^ School of Life Sciences, Anhui Agricultural University, Hefei, China; ^4^ State Key Laboratory of Subtropical Silviculture, Zhejiang A&F University, Hangzhou, China; ^5^ Zhejiang Provincial Key Laboratory of Resources Protection and Innovation of Traditional Chinese Medicine, Zhejiang A&F University, Hangzhou, China

**Keywords:** *LsiSOD* genes, phylogenetic analysis, stress response, duplication events, expression pattern

## Abstract

Superoxide dismutase (SOD) is an important enzyme that serves as the first line of defense in the plant antioxidant system and removes reactive oxygen species (ROS) under adverse conditions. The SOD protein family is widely distributed in the plant kingdom and plays a significant role in plant growth and development. However, the comprehensive analysis of the *SOD* gene family has not been conducted in Cucurbitaceae. Subsequently, 43 *SOD* genes were identified from Cucurbitaceae species [*Citrullus lanatus* (watermelon), *Cucurbita pepo* (zucchini), *Cucumis sativus* (cucumber), *Lagenaria siceraria* (bottle gourd), *Cucumis melo* (melon)]. According to evolutionary analysis, *SOD* genes were divided into eight subfamilies (I, II, III, IV, V, VI, VII, VIII). The gene structure analysis exhibited that the *SOD* gene family had comparatively preserved exon/intron assembly and motif as well. Phylogenetic and structural analysis revealed the functional divergence of Cucurbitaceae *SOD* gene family. Furthermore, microRNAs 6 miRNAs were predicted targeting 3 *LsiSOD* genes. Gene ontology annotation outcomes confirm the role of *LsiSODs* under different stress stimuli, cellular oxidant detoxification processes, metal ion binding activities, SOD activity, and different cellular components. Promoter regions of the *SOD* family revealed that most *cis*-elements were involved in plant development, stress response, and plant hormones. Evaluation of the gene expression showed that most *SOD* genes were expressed in different tissues (root, flower, fruit, stem, and leaf). Finally, the expression profiles of eight *LsiSOD* genes analyzed by qRT-PCR suggested that these genetic reserves responded to drought, saline, heat, and cold stress. These findings laid the foundation for further study of the role of the *SOD* gene family in Cucurbitaceae. Also, they provided the potential for its use in the genetic improvement of Cucurbitaceae.

## Introduction

In natural conditions, plants are often susceptible to drought, salt, extreme temperatures, heavy metals, and other stresses that have a significant impact on the growth, development, and production of plants (Mittler and Blumwald, 2010; Cramer et al., 2011). When a plant is stressed, it adjusts its homeostatic apparatus by producing more reactive oxygen species (ROS) in its cells and ROS, toxic-free radicals produced under stress by plant cells that can oxidize the protein, destroy the cell membrane, and cause DNA damage (Lee et al., 2007; Karuppanapandian et al., 2011; Feng et al., 2015). These stresses inevitably accompany the development of ROS, including hydroxyl radicals (OH), superoxide anion radicals (O_2_^−^), peroxide radicals (HOO^−^), hydrogen peroxide (H_2_O_2_), and singlet oxygen (^1^O_2_), which cause damage to the cell membrane, peroxidization and deterioration of macromolecules, and ultimately lead to the death of cells. ROS are also considered signaling molecules in different organisms and can affect various physiological processes in plants, For example, some prominent active oxygen scavengers can resist environmental stresses by regulating the expression of enzyme reaction family genes such as superoxide dismutase (SOD), catalase (CAT), peroxidase (POD), glutathione peroxidase (GPX), and peroxidase (PrxR) (Mittler et al., 2004; Ahmad et al., 2010; Bafana et al., 2011; Filiz and Tombuloğlu, 2015). Mainly, ROS are formed in the apoplast, mitochondria, plasma membrane, chloroplast, peroxisomes, endoplasmic reticulum, and cell walls (Mittler, 2017; Hasanuzzaman et al., 2020). Therefore, to manage ROS noxiousness, plants have established well-organized and composite antioxidant defense systems, including numerous non-enzymatic and enzymatic antioxidants.

SOD is a type of metal enzyme that is first found in bovine red blood cells and then characterized in bacteria, vertebrates, and higher plants (Mann and Keilin, 1938; Rabinowitch and Sklan, 1980; Tepperman and Dunsmuir, 1990; Kim et al., 1996; Zelko et al., 2002). Researchers of different studies have found that SODs can catalyze superoxide O_2_ to disproportionate into O_2_ and H_2_O_2_ (McCord and Fridovich, 1969; Tepperman and Dunsmuir, 1990). SODs are detected in plants in roots, fruits, leaves, and seeds, which provide necessary protection for cells against oxidative stress (Tepperman and Dunsmuir, 1990). As per the binding pattern of metallic cofactors that cooperate with vigorous sites, *SOD* genes are categorized further into four groups: 1) iron *FeSODs*, 2) copper/zinc *Cu/ZnSODs*, 3) manganese *MnSODs*, and 4) nickel *NiSODs* (Mittler et al., 2004; Feng et al., 2016; Yan et al., 2016; Su et al., 2021). The different subtypes of *SODs* have almost comparable functions. However, they have different metallic cofactors and amino acid sequencing, *in vitro* subcellular location and crystal structure, and different hydrogen peroxide sensitivity (Abreu and Cabelli, 2010; Yan et al., 2016). These SODs are distributed in cell compartments individually and play an essential role in oxidative stress (Alscher et al., 2002). Among the *SODs*, *Cu/ZnSODs* are predominantly distributed in the chloroplast, extracellular space, and cytoplasm, and are present in certain bacteria and all eukaryotes, while *MnSODs* mostly present in plant mitochondria (Pilon et al., 2011; Feng et al., 2015; Song et al., 2018; Wang et al., 2018; Hu et al., 2019; Verma et al., 2019; Lu et al., 2020; Silva et al., 2020). *MnSODs* in the plant genome play a role in eliminating ROS from mitochondria (Møller, 2001). *FeSODs* are primarily distributed in protozoa, prokaryotes, cytoplasms, and plant chloroplasts, while *NiSODs* are found in *Streptomyces* and also in some cyanobacteria, but not in plants (Youn et al., 1996; Wuerges et al., 2004; Miller, 2012).

Recent studies have shown that SODs can protect plants from abiotic stress such as heat, drought, cold, abscisic acid, salt, and ethylene (Wang et al., 2004; Pilon et al., 2011; Asensio et al., 2012; Feng et al., 2015). Several studies have shown that the *SOD* genes can be induced and transcribed in different plants under various stress conditions, such as heat, drought, cold, salt, osmotic stress, oxidative stress, and hormonal signal transduction (Wang et al., 2004; Pilon et al., 2011; Feng et al., 2015; Feng et al., 2016; Yan et al., 2016). *SOD* gene family under different hormones and abiotic stress conditions in rapeseed (Su et al., 2021), *Salvia miltiorrhiza* (Han et al., 2020), *Zostera marina* (Zang et al., 2020), and *Hordeum vulgare* (Zhang et al., 2021) were recently published articles. Furthermore, diverse forms of *SOD* genes display different expression patterns under different stress conditions. In tomatoes, for example, *SlSOD1* is the only significantly upregulated gene in the nine *SlSOD* genes, while *SlSOD*2, *SlSOD*5, *SlSOD*6, and *SlSOD*8 are regulated by salt stress. However, the expression levels of four “*SlSOD*2, *SlSOD*5, *SlSOD*6, and *SlSOD*8” genes are found high during imposed drought environment (Feng et al., 2016). Furthermore, the expression patterns of the same type of *SOD* gene were different under stress. For example, the studies found that there was no change in the expression of *MnSODs* in *Arabidopsis* under oxidative stress, and the researchers found that there was a significant change in the expression of *MnSODs* in peas, wheat, and *Zostera marina* under salt stress (Gómez et al., 1999; Wu et al., 1999; Baek and Skinner, 2003; Zang et al., 2020). These results suggest that different *SOD* genes have different expression patterns under different environmental stresses. Furthermore, researchers have also discovered that alternative splicing and miRNAs may be involved in the regulation of *SOD* expression (Srivastava et al., 2009; Lu et al., 2011). Until now, the *SOD* gene family has been described in many plant species, including *Arabidopsis*, *Sorghum*, *Musa acuminata*, *Phaseolus vulgaris*, and *Populus* (Kliebenstein et al., 1998; Zelko et al., 2002; Ballal and Manna, 2009; Fernández-Ocaña et al., 2011; Lin and Lai, 2013; Molina-Rueda et al., 2013; Filiz et al., 2014; Cui et al., 2015; Feng et al., 2015; Feng et al., 2016; Ho et al., 2017; Verma et al., 2019). Cucurbitaceae is a significant economic and nutritional crop in the world and *SOD* gene exists as a superfamily. The phylogeny line, genome circulation, gene assembly, preserved patterns, and expression profiles of these genes in different tissues have been thoroughly analyzed, laying the foundation for functional identification of SOD genes in Cucurbitaceae.

## Materials and Methods

### Retrieval of *SOD* Gene Family in Five Cucurbitaceae Species

To study the *SOD* gene family of cucurbit plants, the whole genome of individual species of Cucurbitaceae was searched by the Blastp search method and *Arabidopsis* sequence of SOD used as the query (Kliebenstein et al., 1998). Genomic, protein, and CDS (coding DNA sequence) sequences of *SOD* gene family have been identified and downloaded from the Cucurbitaceae database (CuGenDB) (http://cucurbitgenetics.org/) (Zheng et al., 2019) Subsequently, we used two methods to identify SOD genes in five Cucurbitaceae species, i.e., BLASTP (protein blast) and the hidden Markov model (HMM). For BLASTP, we used eight *A. thaliana SODs* (AT1G08830.1/*AtCSD1*, AT2G28190.1/*AtCSD2*, AT5G18100.1/*AtCSD3*, AT4G25100.1/*AtFSD1*, AT5G51100.1/*AtFSD2*, AT5G23310.1/*AtFSD3*, AT3G10920.1/*AtMSD1*, and AT3G56350.1/*At00MSD2*) amino acid sequences as a query with an e-value set to 1e−5. The amino acid sequences of eight *AtSODs* were obtained from the TAIR *Arabidopsis* genome database (http://www.arabidopsis.org/). The database web resources SMART (http:/smart.embl-heidelberg.de/) (Schultz et al., 1998) and the Pfam protein domain database (http://pfam.xfam.org/) were used to scan certain amino acid sequences conserved domain SOD_Cu (PF00080.21) and SOD_Fe_C (PF02777.19). The ProtParam software (http:/web.expasy.org/ProtParam) (Gasteiger et al., 2005) was used to evaluate the physiochemical properties (molecular weight, amino acid length, and isoelectric point) of each SOD protein.

### Phylogenetic Analyses of *SOD* Gene Family

All SOD full-length protein sequences of the five Cucurbitaceae species [*Citrullus lanatus* (watermelon), *Cucurbita pepo* (zucchini), *Cucumis sativus* (cucumber), *Lagenaria siceraria* (bottle gourd), *Cucumis melo* (melon)] including seven other plant species were aligned using clustalX software with default parameters (1,000 bootstraps, pairwise deletion) (Thompson et al., 1997). A phylogenetic tree was constructed with a maximum likelihood method (MLM) by using the online version of the IQ-TREE software (http:/iqtree.cibiv.univie.ac.at). Finally, the phylogenetic tree was visualized through online iTOL software (https://itol.embl.de/) (Letunic and Bork, 2019; Manzoor et al., 2021a).

### Chromosomal Distribution of *SOD* Genes

The chromosomal localizations of Cucurbitaceae were obtained from CuGenDB database (http://cucurbitgenetics.org/). For chromosome mapping of *SOD* gene, map inspect tool (http://mapinspect.software.com) was used. The aforementioned description is used to map the distribution of *SOD* gene throughout the Cucurbitaceae family individual species used in this study (Hu et al., 2016).

### Intron/Exon Structure and Conserved Motif Analysis of Protein

The genome sequence and the coding sequence of the *SOD* gene were downloaded from the genome of the individual species of the Cucurbitaceae family from CuGenDB database. The Gene Structure and Display Server (GSDS) (http:/gsds.cbi.pku.edu.cn/) (Hu et al., 2015) was used to evaluate intron distribution dynamics and the splicing mechanism of each *SOD* gene. The Multiple Expectation Maximization for Motif Elicitation (MEME) software (Bailey et al., 2009) identified the conserved SOD protein motif (http:/meme-suite.org/tools/meme). Finally, the subcellular localization was predicted using WoLF PSORT (http://wolfpsort.org) (Horton et al., 2007).

### Prediction of the *SOD Cis*-Regulatory Elements in the Promoter

For the prediction of regulatory elements on the promoter regions of *CuSODs*, 1,500 kb upstream DNA sequence of each *SOD* gene was collected from all *SOD* genes using the online web server PlantCARE (http:/bioinformatics.psb.ugent.be/webtools/PlantCARE/html/) (Higo et al., 1999; Lescot et al., 2002).

### Gene Duplications and Collinearity Relationship Analysis

Multiple collinearity scan toolkit (MCScanX) was used for collinearity analysis with BLASTP (E < 1e−5) against five Cucurbitaceae species (Wang et al., 2012; Qiao et al., 2018). Different modes of duplications (WGD/segmental, dispersed, proximal, and tandem duplications) among five Cucurbitaceae species (*Citrullus lanatus*, *Cucurbita pepo*, *Cucumis sativus*, *Lagenaria siceraria*, *Cucumis melo*) were used. Gene duplications and collinearity relationships were visualized by using TBtools (Chen et al., 2020) and circos software.

### Gene Ontology Annotation and MicroRNA Target Site Analysis

We used the CELLO v.2.5 software functional annotation platform to determine the Cucurbitaceae *SOD* gene’s functional Classification. CELLO (http://cello.life.nctu.edu.tw/site) platform connects the genes with GO terms through hierarchical vocabularies (Conesa and Götz, 2008). Functional enrichment analysis of *SOD* genes was performed using DAVID online tools (DAVID 6.8; http://david.ncifcrf.gov/) (Huang et al., 2009). The GO terms were classified into three categories: biological process (BP), cellular component (CC), and molecular function (MF). The upregulated *SOD* genes and downregulated *SOD* genes were entered separately and *p* <0.01 was considered to indicate a statistically significant difference.

To determine the miRNA-mediated posttranscriptional regulation of *SOD*s, we searched the 5′ and 3′ untranslated regions (UTRs), and the coding regions, of the *SODs* for target sites of *Lagenaria siceraria* miRNAs obtained from various databases and published articles on the psRNA Target server using default parameters (Dai et al., 2018).

### Plant Materials and Abiotic Stresses

The *L. siceraria* plants were planted in the growth chamber; seeds (variety: Winall 808) were provided by The National Engineering Laboratory of Crop Resistance Breeding, School of Life Sciences, Anhui Agricultural University, Hefei, China. To examine the behavior of the *SOD* genes under abiotic stress, seedlings of 2-week-old plants were carefully watered and grown under 24 h/18 h light and 16 h/8 h dark conditions in a growth chamber before different stress inductions. However, seedlings were later transferred to the growth chamber at 50°C under normal lighting conditions during the heat treatment. The plant was treated for salt and drought resistance on the Murashige and Skoog (MS) liquid media containing 300 mM of PEG-6000. For the cold treatment, seedlings in the growth chamber were transferred to 4°C under light conditions (Hu et al., 2016). After treatment, the leaves were collected at 0, 4, 8, 16, and 24 h, and instantly frozen in liquid nitrogen immediately, and the sample was obtained to determine the transcription level of the *SOD* gene family in treated plant seedlings. Finally, all samples were instantly frozen in liquid nitrogen and stored at −80°C until further use.

### RNA Isolation and Quantitative Real-Time PCR Reaction

RNA extraction reagent kit (Trizol) was purchased from Hua and Maike Biotechnology Co. Ltd., Beijing, China. Total RNA was extracted following the manufacturer’s instructions. The RNA reverse transcription kit (TaKaRa Company, Japan) and Fluorescent Quantitative Reagent SYBR Green Master (Roche, United States) were used, and qRT-PCR was performed using a previously described method (Zhou et al., 2014). Gene-specific primers of *LsiSOD*s and *Lsi*-actin for the qRT-PCR system were designed by using GenScript server (www.genscript.com) and synthesized by Sangon Biotech (Shanghai) (Supplementary Table S1).

Actin gene of *L. siceraria* was used as an endogenous control to detect the relative expression of *LsiSOD* genes based on previous studies (Zhang et al., 2018). Primers used are given in Supplementary Table S1. qRT-PCR reactions were performed in biological triplicates. The relative expression level was calculated by 2^−∆Ct^ method and statistical analyses were measured using Microsoft Office 2010.

## Results

### Genome-Wide Characterization of *SOD* Genes in Five Cucurbitaceae Species

In total, 43 *SODs* were identified in five Cucurbitaceae species from CuGenDB, and 8–10 genes were retrieved for individual species such as *Citrullus lanatus* (8 *SODs*), *Cucurbita pepo* (10 *SODs*), *Cucumis sativus* (9 *SODs*), *Lagenaria siceraria* (8 *SODs*), and *Cucumis melo* (8 *SODs*) (Supplementary Table S2). The detailed information of Cucurbitaceae species (molecular weight, starting and ending point, and theoretical p*I*) of the protein are listed in Table 1, and all protein sequences are shown in Supplementary Table S10.

**TABLE 1:** Physio-chemical characteristics of 43 *SOD* identified genes and sequence information in five species of Cucurbitaceae family.

Entry ID	Pfam domain		Exon	Intron	Protein						DNA	
					Localization by PC	MW kDa^1^	AA^2^	p*I* ^3^	pH^4^	Gray^5^	MW Da^6^	Length^7^
ClaSOD7	IMA, IMC	PF00081, PF02777	8	7	Chloroplast	34.56	304	6.17	6.19	−0.757	1,415,651.18	4,585
ClaSOD8	HMA.CZ	PF00080	7	6	Chloroplast	35.01	330	5.26	5.26	−0.083	1,453,499.90	4,704
CpSOD1	CZ	PF00080	5	4	Chloroplast	12.45	122	6.71	7.32	−0.298	82,357.07	960
CpSOD2	CZ	PF00080	6	5	Cytoplasm	17.14	162	5.18	5.18	−0.255	1,096,062.63	3,657
CpSOD3	CZ	PF00080	2	1	Chloroplast	53.46	52	8.40	9.54	−0.010	20,704.82	246
CpSOD4	IMA, IMC	PF00081, PF02777	8	7	Chloroplast	33.36	295	5.51	5.51	−0.603	842,465.78	2,730
CpSOD5	IMA, IMC	PF00081, PF02777	6	5	Mitochondrion	26.23	235	7.94	8.07	−0.274	1,150,425.35	3,733
CpSOD6	CZ	PF00080	8	7	Chloroplast	22.50	221	6.02	6.03	0.108	1,126,708.80	3,645
CpSOD7	CZ	PF00080	6	5	Chloroplast	34.22	323	5.37	5.24	−0.053	347,046.97	4,122
CpSOD8	CZ	PF00080	7	6	Chloroplast	15.28	152	5.28	5.28	−0.117	848,531.63	2,741
CpSOD9	CZ, Thio	PF00080	5	4	Cytoplasm	59.17	530	9.73	9.73	−0.168	995,717.59	3,226
CpSOD10	IMA, IMC	PF00081, PF02777	6	5	Mitochondrion	23.79	216	7.98	8.74	−0.197	229,375.36	2,730
CsaSOD1	IMA, IMC	PF00081, PF02777	6	5	Mitochondrion	26.88	242	7.88	8.01	−0.245	1,145,395.55	3,707
CsaSOD2	IMA, IMC	PF00081, PF02777	8	7	Mitochondrion	30.85	267	6.79	6.86	−0.390	2,029,893.05	6,567
CsaSOD3	CZ	PF00080	7	6	Chloroplast	15.26	152	5.44	5.44	−0.140	1,369,045.72	4,434
CsaSOD4	CZ	PF00080	7	6	Chloroplast	15.40	152	4.97	4.97	−0.121	1,446,146.27	4,677
CsaSOD5	CZ, HMA	PF00080	5	4	Nucleus	22.09	223	9.83	10.44	−0.417	315,861.52	3,708
CsaSOD6	IMA, IMC	PF00081, PF02777	8	7	Chloroplast	36.38	318	5.78	5.78	−0.825	1,242,734.18	4,017
CsaSOD7	CZ	PF00080	7	6	Cytoplasm	15.87	157	6.53	6.57	−0.154	1,315,300.39	4,267
CsaSOD8	CZ	PF00080	8	7	Chloroplast	22.62	223	5.87	5.88	0.178	1,851,704.38	5,987
CsaSOD9	CZ	PF00080	3	2	Nucleus	86.82	73	5.80	5.85	−0.203	26,119.11	316
LsiSOD1	AP2, CZ	PF00080	8	7	Chloroplast	42.32	397	8.51	8.52	−0.376	3,503,807.65	11,347
LsiSOD2	IMA, IMC	PF00081, PF02777	8	7	Mitochondrion	32.45	292	6.67	5.36	−0.314	1,577,617.15	5,112
LsiSOD3	IMC, IMA	PF00081, PF02777	6	5	Mitochondrion	31.25	274	8.65	8.66	−0.369	1,709,879.76	5,529
LsiSOD4	CZ	PF00080	7	6	Chloroplast	15.07	152	5.59	5.59	−0.030	1,095,314.26	3,557
LsiSOD5	IMA, IMC	PF00081, PF02777	6	5	Chloroplast	32.29	286	6.11	5.90	−0.753	1,752,081.95	5,669
LsiSOD6	HMA, CZ	PF00080	6	5	Chloroplast	36.61	344	6.84	7.01	−0.125	1,422,371.22	4,647
LsiSOD7	CZ	PF00080	10	9	Chloroplast	32.46	239	5.90	5.90	0.030	1,636,024.86	5,283
LsiSOD8	CZ	PF00080	7	6	Chloroplast	17.93	171	5.35	6.12	−0.264	1,418,129.87	4,591
MelSOD1	IMA, IMC	PF00081, PF02777	8	7	Mitochondrion	30.82	267	8.24	8.50	−0.378	1,216,363.52	3,937
MelSOD2	CZ	PF00080	7	6	Chloroplast	17.59	172	5.61	6.16	−0.091	1,452,368.96	4,704
MelSOD3	CZ	PF00080	6	5	Cytoplasm	13.73	134	5.48	8.28	−0.171	1,817,515.13	5,882
MelSOD4	IMA, IMC	PF00081, PF02777	6	5	Chloroplast	36.94	347	8.49	5.48	−0.061	1,364,456.23	4,424
MelSOD5	IMA, IMC	PF00081, PF02777	7	6	Chloroplast	33.07	291	6.15	6.57	−0.829	1,331,923.50	4,313
MelSOD6	CZ	PF00080	7	6	Cytoplasm	15.90	157	6.53	5.61	−0.116	960,111.72	3,110
MelSOD7	CZ	PF00080	7	6	Chloroplast	22.01	217	5.78	8.65	0.070	996,665.45	3,222
MelSOD8	IMA, IMC	PF00081, PF02777	5	4	Mitochondrion	30.89	278	8.65	5.88	0.003	802,622.21	2,607

1, molecular weight of protein; 2, amino acid; 3, protein isoelectric point; 4, pH; 5, GRAVY; 6, molecular weight of DNA; 7, length. CZ, copper/zinc superoxide dismutase (PF00080) (SODC); IMA, iron/manganese superoxide dismutase alpha-hairpin domain (PF00081); IMC, iron/manganese superoxide dismutase (PF02777), C-terminal domain; PC, ProtComp9.0 server.

In *Citrullus lanatus*, we detected eight *SOD* genes, containing five copper/zinc *SODs* and three iron/manganese *SODs*. The molecular weight, length, and p*I* of SOD protein were 15.12–35.01 kDa, 152–330 amino acids, and 5.31–7.95, respectively: five *Cu/ZnSODs* (*ClaSOD*1, *ClaSOD*2, *ClaSOD*3, *ClaSOD*6, *ClaSOD*8) were acidic in nature and one *MnSOD* (*ClaSOD*5) was basic, and in the two *FeSODs*, *ClaSOD*7 was acidic and *ClaSOD*4 was slightly basic. Subcellular localization prediction results showed that six SODs (*ClaSOD1*, *ClaSOD2*, *ClaSOD3*, *ClaSOD6*, *ClaSOD7*, *ClaSOD8*) of *Cu/Zn SODs* were in the chloroplast, and *Cla*-*SOD*4 and *Cla-SOD*5 were localized to the mitochondria.

In *Cucurbita pepo*, ten *SOD* genes were detected, including seven *Cu/ZnSODs* and three Fe/Mn-SODs; the molecular weight, length, and p*I* values of SOD proteins were within the ranges of 15.28–59.17 kDa, 52–530 amino acids, and 5.18–9.73, respectively. The subcellular localization prediction showed that *Cu/ZnSODs CpSOD1*, *CpSOD3*, *CpSOD4*, *CpSOD6*, and *CpSOD7* were localized to the chloroplast, and two *Cu/ZnSODs*, *CpSOD*2 and *CpSOD*9, were localized to the cytoplasm. Furthermore, *CpSOD*5 and *CpSOD*10 were localized to the mitochondrion.

In *Cucumis sativus*, nine *SODs* were retrieved—six were *Cu/ZnSODs* and three were *Fe/MnSODs*. The molecular weight, length, and p*I* values of SOD proteins were within the range of 15.26–86.82, while amino acids range from 73 to 318 and 4.97–9.83 kDa, respectively. *CsaSOD2*, *CsaSOD3*, *CsaSOD4*, *CsaSOD6*, *CsaSOD7*, *CsaSOD8,* and *CsaSOD9* were acidic and *CsaSOD1* and *CsaSOD5* were basic. In the prediction of subcellular localization, *CsaSOD*1 and *CsaSOD*2 were localized to the mitochondrion. *CsaSOD3*, *CsaSOD4*, *CsaSOD6*, and *CsaSOD8* are localized to the chloroplast; *CsaSOD5* and *CsaSOD9* are in the nucleus; and *CsaSOD7* is localized to the cytoplasm.

In *Lagenaria siceraria*, eight *SODs* were detected collectively, including five copper/zinc SODs and three iron/manganese *SODs*. The molecular weight, length, and p*I* of SOD protein were 15.07–42.32 kDa, 152–397 amino acids, and 5.35–8.65, respectively. *LsiSOD1* and *LsiSOD3* of Cu/Zn-SODs are basic and *LsiSOD6* is slightly basic; *LsiSOD2*, *LsiSOD4*, *LsiSOD5*, *LsiSOD7*, and *LsiSOD9* are acidic. Subcellular localizations of *LsiSOD1*, *LsiSOD4*, *LsiSOD5*, *LsiSOD6*, *LsiSOD7*, and *LsiSOD8* are found in the chloroplast while *LsiSOD*2 and *LsiSOD*3 are in the mitochondrion.

In *Cucumis melo*, a total of eight *SODs* were identified, including four *Cu/ZnSODs* and four *Fe/MnSODs*, and the SOD protein’s molecular weight, length, and p*I* values were observed within the ranges of 15.90–36.95 kDa, 134–347 amino acids, and 5.16–8.49, respectively; three SODs such as *MelSOD1*, *MelSOD4*, and *MelSOD8* are basic, and *MelSOD2*, *MelSOD3*, *MelSOD5*, *MelSOD6*, and *MelSOD7* are acidic. The prediction of subcellular localizations showed that *MelSOD1* and *MelSOD8* are in the mitochondrion, and *MelSOD3* and *MelSOD7* are in the cytoplasm. *MelSOD2*, *MelSOD4*, and *MelSOD5* are in the chloroplast (Table 1).

### Structural Phylogenetic Analysis of *SOD* Genes Family in Five Cucurbitaceae Species

**FIGURE 1:**
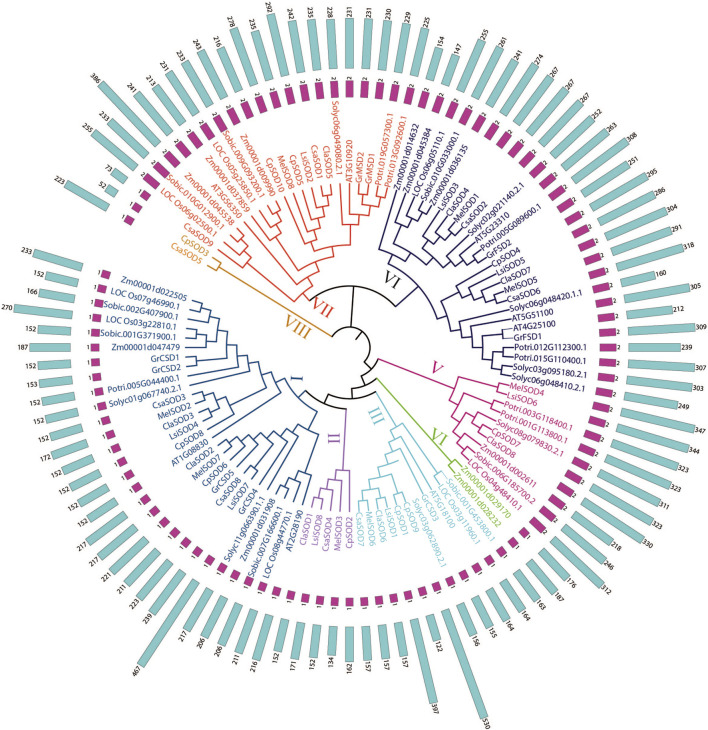
Phylogenetic tree of 106 SOD proteins from *Lagenaria siceraria* and other plants including 13 plant species—*Arabidopsis thaliana*, *Lagenaria siceraria*, *Citrullus lanatus*, *Cucurbita pepo*, *Cucumis sativa*, *Cucumis melo*, *Gossypium arboretum*, *Solanum lycopersicum*, *Zea mays*, *Solanum tuberosum*, *Sorghum bicolor*, *Populus trichocarpa*, and *Oryza sativa*. Protein sequences were aligned using ClustalW2 sequence alignment program and the phylogenetic tree was constructed by software MEGA7.

To define the evolutionary relationship of SODs in different plant species, we constructed a phylogenetic tree of SODs based on the full length of protein sequences and divided them into eight groups. A total of 106 SOD proteins were obtained from 13 plant species including *Arabidopsis thaliana*, *Lagenaria siceraria*, *Citrullus lanatus*, *Cucurbita pepo*, *Cucumis sativa*, *Cucumis melo*, *Gossypium arboretum*, *Solanum lycopersicum*, *Zea mays*, *Solanum tuberosum*, *Sorghum bicolor*, *Populus trichocarpa*, and *Oryza sativa*. Three methods, maximum likelihood (ML), minimal evolution (ME), and maximum parsimony (MP), yielded nearly identical phylogenetic trees; therefore, only NJ tree was used for further analyses (Kumar et al., 2016) (Figure 1; Supplementary Figure S2). Based on the phylogenetic tree, the *SOD* genes were divided into five subgroups according to *Arabidopsis* (Silva et al., 2020). However, three unique clades in a phylogenetic tree may have independent evolutionary trajectories from other clades. It is also suggested that these clades may have individual evolution that is different from *Arabidopsis* (Nijhawan et al., 2008; Wei et al., 2012; Manzoor et al., 2021a). According to the bootstrap values quoted on the nodes, topography, and sequence similarities, all identified *SODs* from the Cucurbitaceae species were categorized into eight subfamilies (I–VIII). SOD protein sequences from all Cucurbitaceae species contributed in all subfamilies (Figure 1). These results suggested that there may have been gene loss or gain events that occurred throughout the evolutionary process. The gain and loss of specific *SOD* gene members caused functional divergence.

### Conserved Motif Analysis of *SOD* Gene Family in Five Cucurbitaceae Species

**FIGURE 2:**
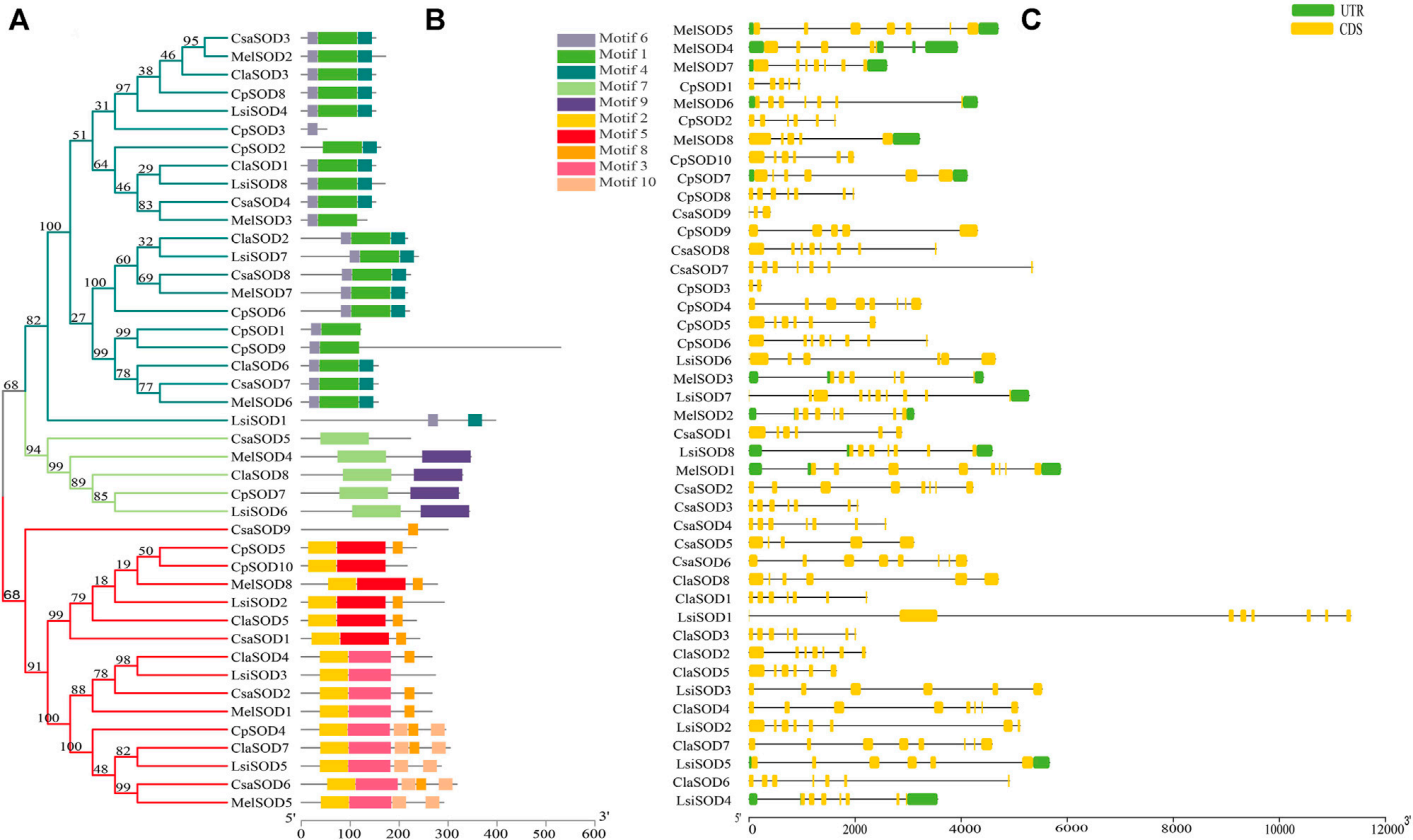
**(A)** The protein structures of SODs based on the presence of conserved motifs were arranged corresponding to the phylogenetic tree. **(B)** All conserved motifs of the SOD proteins were identified by the MEME program. Different motifs are highlighted with different colored boxes with numbers 1 to 10. **(C)** Gene structures of SODs exons are shown as yellow boxes, introns are shown as thin black lines, and UTRs are shown as blue boxes.

The conserved motif analysis of the *SOD* family supported the classification and evolutionary relationships of the five Cucurbitaceae species *SOD* genes. In total, 20 motifs were detected from 43 *SOD* genes in *Citrullus lanatus*, *Cucurbita pepo*, *Cucumis sativus*, *Lagenaria siceraria*, and *Cucumis melo*. All *SOD* genes contained at least two motifs except *CpSOD*3 and *CsaSOD*5, which contain only one motif (Figure 2B): *Cu/ZnSOD* and *Fe/MnSODs*. Besides, *FeSODs* and *MnSODs* were clustered in the same group and belonged to a similar subcluster, while the *Cu/ZnSOD* was clustered in a different group. A similar cluster distribution was detected in the SOD proteins of each species, indicating that these *SOD* genes are highly conserved in different plants (Figure 2A). In Cucurbitaceae species, 5–9 exons were detected in *SOD* genes by comparing the genomic DNA and CDS sequences using the GSDS 2.0 utility (Figure 2C). However, differences were observed in the size and number of exons/introns in Cucurbitaceae. In the organization of exons/introns, a high degree of conservation has been observed, which is consistent with the high degree of similarity found by multiple alignments between protein sequences, which gives high similarities between them. The evolutionary analysis suggested that structural gene diversity is the primary source for the evolution of multigene families (Xu et al., 2012; Manzoor et al., 2021b).

*C. lanatus* contains 6–8 exons and *C. pepo* contains 3–9 exons; interestingly, *CpSOD3* contains 2 exons with 1 intron. *C. sativus* contains 5–8 exons while *CsaSOD9* has 3 exons and 2 introns. Both *CpSOD3* and *CsaSOD9* genes are the smallest genes among all 43 SOD genes identified in five Cucurbitaceae species. *C. melo* consists of 5–8 exons in which *MelSOD8* has 5 exons and 4 introns. *L. siceraria* contains 6–10 exons; interestingly, *LsiSOD1* and *LsiSOD*2 comprise 8/7 exons/introns. On the other hand, *LsiSOD3*, *LsiSOD5*, and *LsiSOD6* comprise 6/5 exons/introns that have the upstream and downstream sequence. *LsiSOD4* contains exons/introns (7/6) with upstream and downstream sequences and *LsiSOD7* contains 10 exons with the only downstream sequence. The remaining *LsiSOD8* contains 7 exons and 6 introns with both upstream and downstream sequences.

### Chromosomal Distribution and Promoter Analysis of *SOD* Gene Family

**FIGURE 3:**
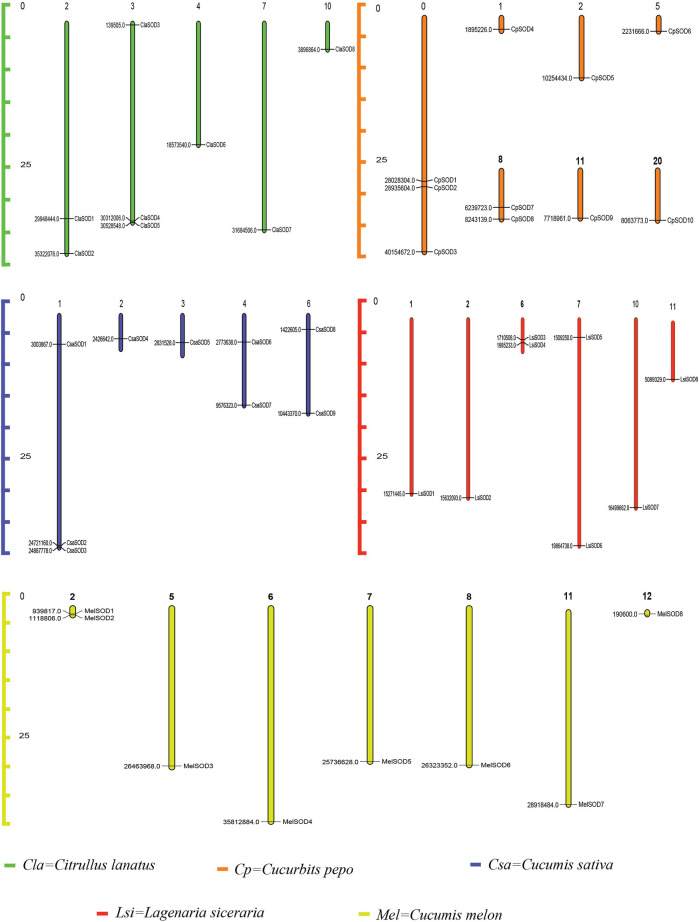
Chromosomal locations of 43 SOD genes in the genome of five species of the Cucurbitaceae family. Watermelon: Cla; melon: Mel; cucumber: Csa; zucchini: Cp; bottle gourd: Lsi. The chromosome numbers are indicated at the top of chromosomes, and the size of the chromosome is represented with a vertical scale. The gene number is located on each chromosome and the left scale is in megabases (Mb).

The chromosomadal distribution of the *SOD* gene family of the Cucurbitaceae species was determined, as shown in Figure 3 (Supplementary Table S3), and detailed data are given in Supplementary Table S4. *C. lanatus* chromosome 2 contains two genes, and chromosome 3 contains three genes, while chromosomes 4, 7, and 10 contain only one gene. In *C. pepo*, chromosome 0 contains three genes; 1, 2, 5, 11, and 20 contain only one gene while chromosome 8 contains 2 genes. In *C. sativus*, chromosome 1 contains three genes, and chromosomes 4 and 6 contain two genes each, while only one gene was found on chromosomes 2 and 3. In *L. siceraria*, chromosomes 1, 2, 10, and 11 contain one gene, while chromosomes 6 and 7 contain two genes, respectively. *C. melo* contains two genes on chromosome 2, while chromosomes 5, 6, 7, 8, 11, and 12 contain only one *SOD* gene.

**FIGURE 4:**
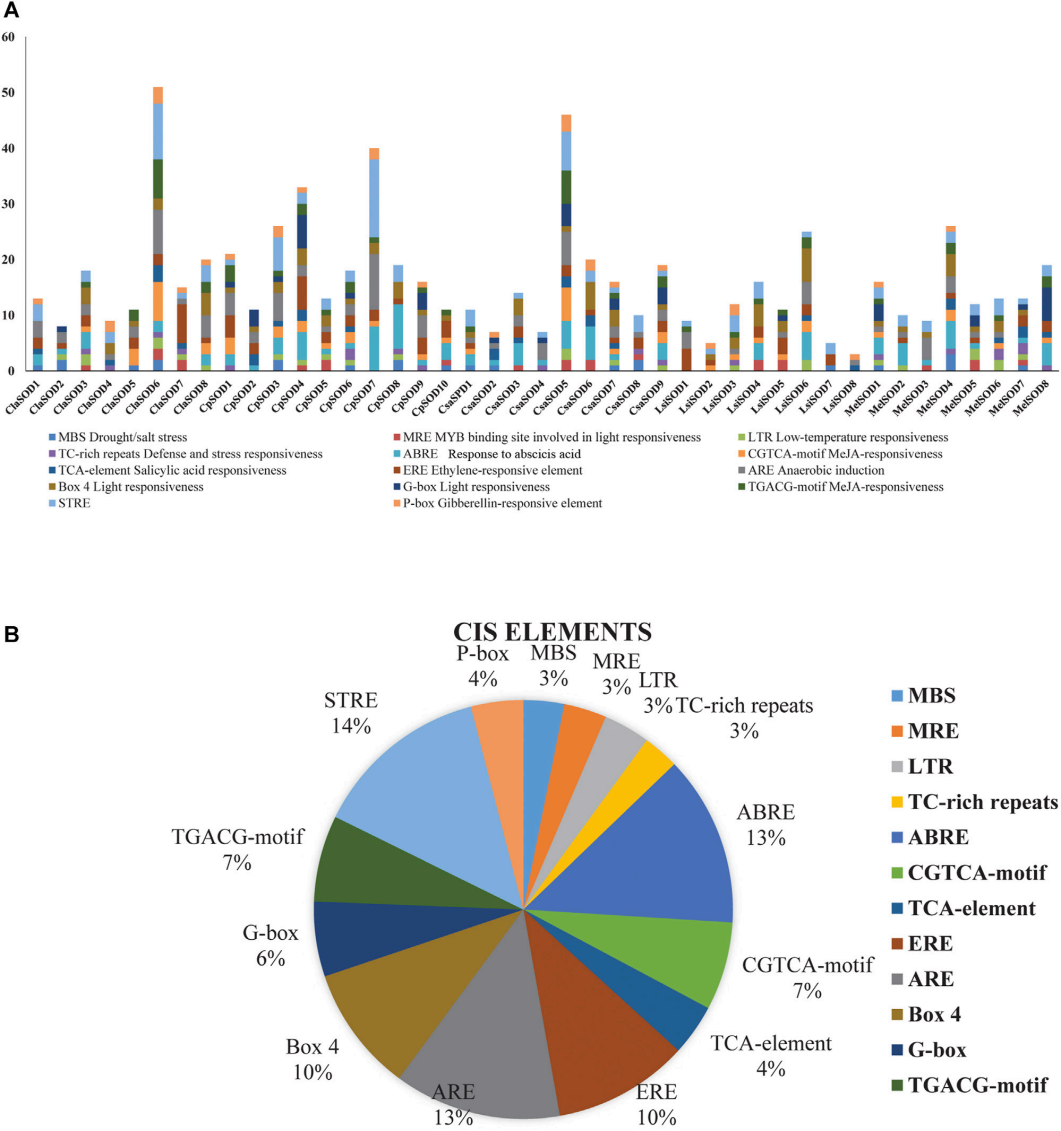
Analysis of *cis*-elements in putative promoters of SOD gene family of five species of Cucurbitaceae family. 1,500 kb upstream of the transcription initiation site, sequences of SOD genes were retrieved and analyzed by PlantCARE. **(A)** Different stress-responsive and hormone-associated elements are identified, and their numbers are plotted on the graph. Various elements in each promoter are coded in different colors according to the legend at the top. **(B)** The size of the pie charts showed the percentage of promoter element in each category.

To clearly understand the function and regulation of SOD proteins, *cis*-elements in the promoter sequence of *SOD* genes in Cucurbitaceae were retrieved. The 1,500-bp upstream region of the start codon of each *SOD* gene was analyzed by using the PlantCARE database. According to the obtained results, the *cis*-elements can be divided into three classes: stress related, light related, and hormone response elements (Figure 4A). Five *cis*-elements of stress response were determined, including LTR, TC rich, MBS, ARE, and box-w1; these elements reflected the response of plants to drought, low temperature, anaerobic induction, stress defense, and fungal inducer. Four hormone-sensitive *cis*-elements (SA, MeJA, GA, and ethylene) were identified, which are associated with ABA, SA, ethylene, and MeJA responses (Figure 4B). On the promoter region of *SOD* gene family of Cucurbitaceae, a considerable number of phyto-response *cis*-elements were detected (Supplementary Table S4). The results suggested that the *cis*-elements of *SOD* promoter had a positive response to abiotic stress and regulation mechanism of plant growth and development.

### Gene Duplication Events and Collinearity Relationships in Five Cucurbitaceae Genomes

**FIGURE 5:**
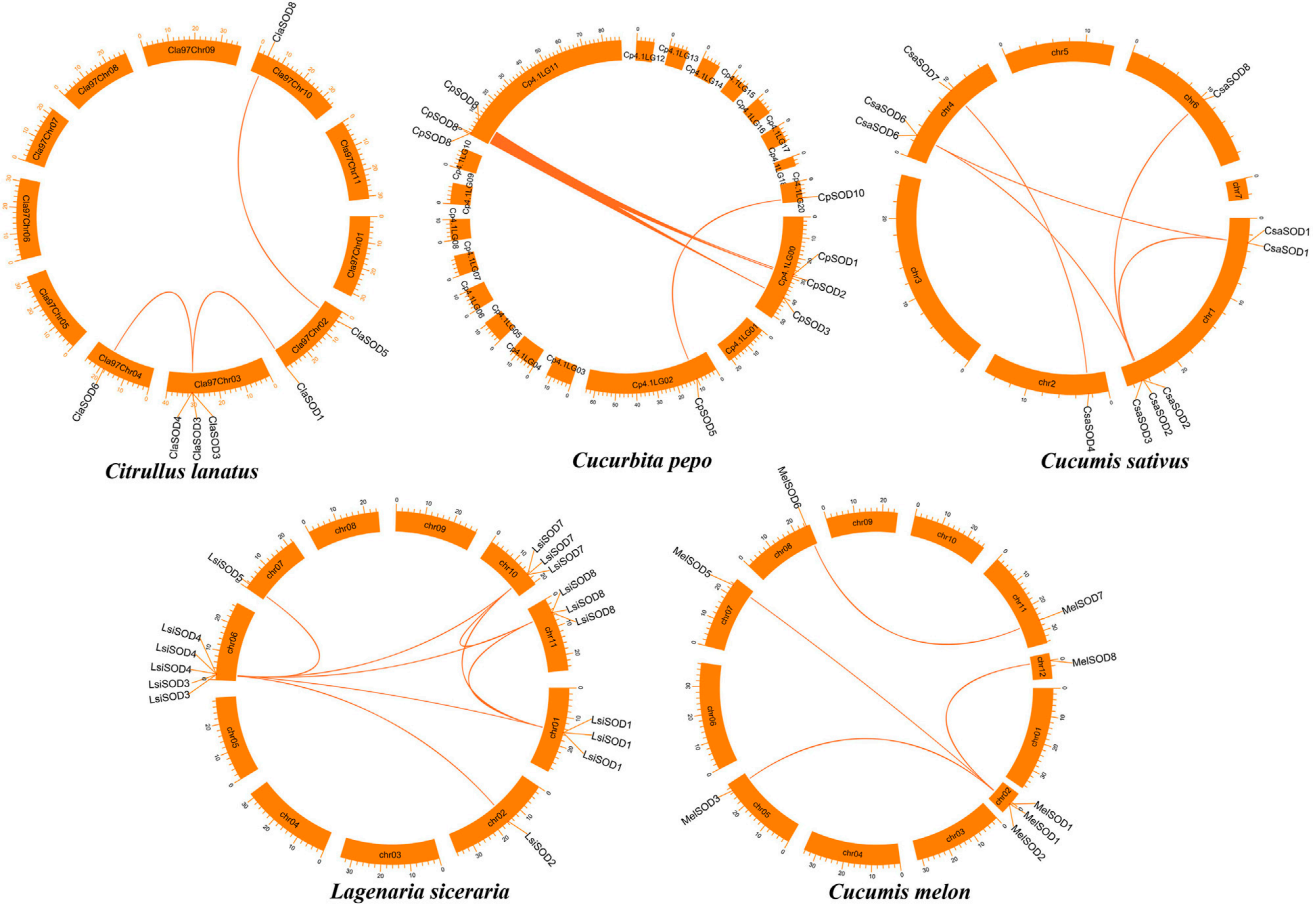
Chromosomal localization and duplication events of five Cucurbitaceae genomes and gene pairs are joined with a colored line.

**FIGURE 6:**
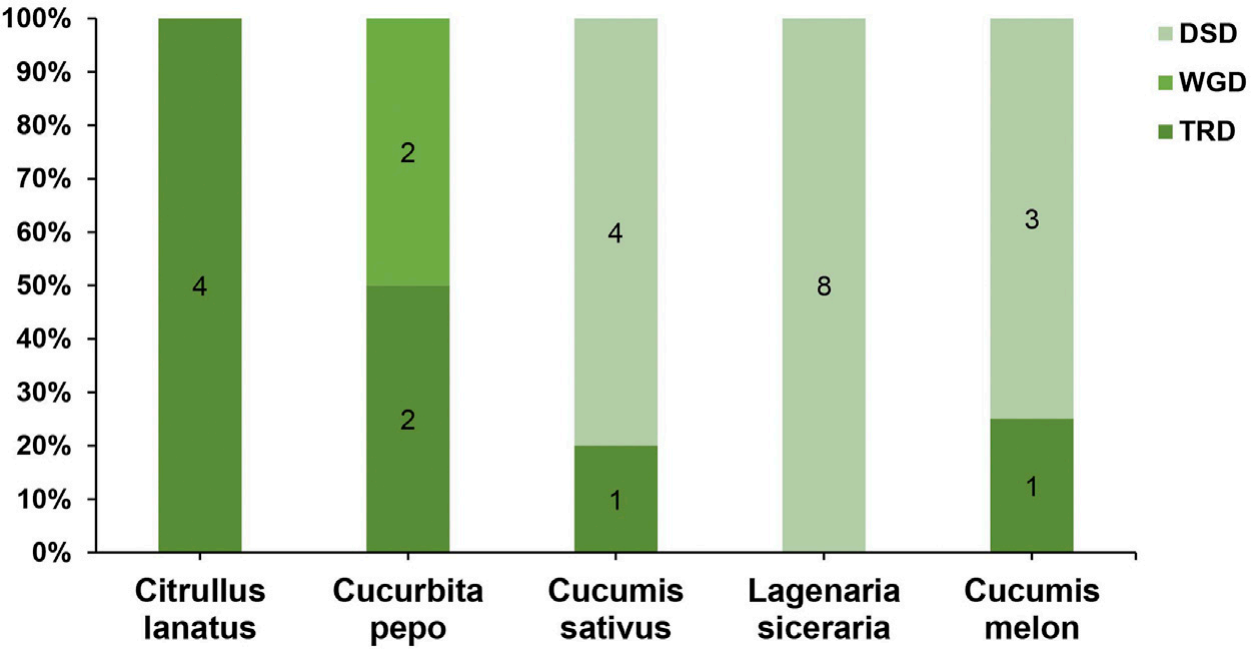
Comparison of gene duplication events in five Cucurbitaceae genomes. WGD, whole-genome duplicates; TRD, transposed duplicates; DSD, dispersed duplicates.

To further understand the evolution and new function of genes, gene duplication events of the *SOD* gene family were identified in five Cucurbitaceae species (*Citrullus lanatus*, *Cucurbita pepo*, *Cucumis sativus*, *Lagenaria siceraria*, *Cucumis melo*). We analyzed three modes of gene duplications in all *SOD* gene families, including transposed duplication (TRD), dispersed duplication (DSD), and whole-genome duplication (WGD). We identified many kinds of gene duplications and their contributions to the expansion of the *SOD* genes. Interestingly, 25 pairs of the duplicated gene were identified in five Cucurbitaceae species, with a maximal number of duplicated gene pairs derived from dispersed duplications (15 genes pair out of 25), followed by transposed duplications (8 genes pair out of 25) and whole-genome duplications (2 genes pair out of 25) showing that the expression of the *SOD* gene family was mainly associated with WGD, TRD, and DSD events (Supplementary Table S5) (Figure 5). These results indicate that DSDs play a vital role in *SOD* gene expansion in *Cucumis sativus*, *Lagenaria siceraria*, and *Cucumis melo*. WGDs might contribute to the expansion of the *SOD* gene family (Supplementary Table S5). Our study showed that duplication events play an important role in *SOD* gene expansion, and TRDs and WGD occurred at high frequency in Cucurbitaceae species (Figure 6).

**FIGURE 7:**
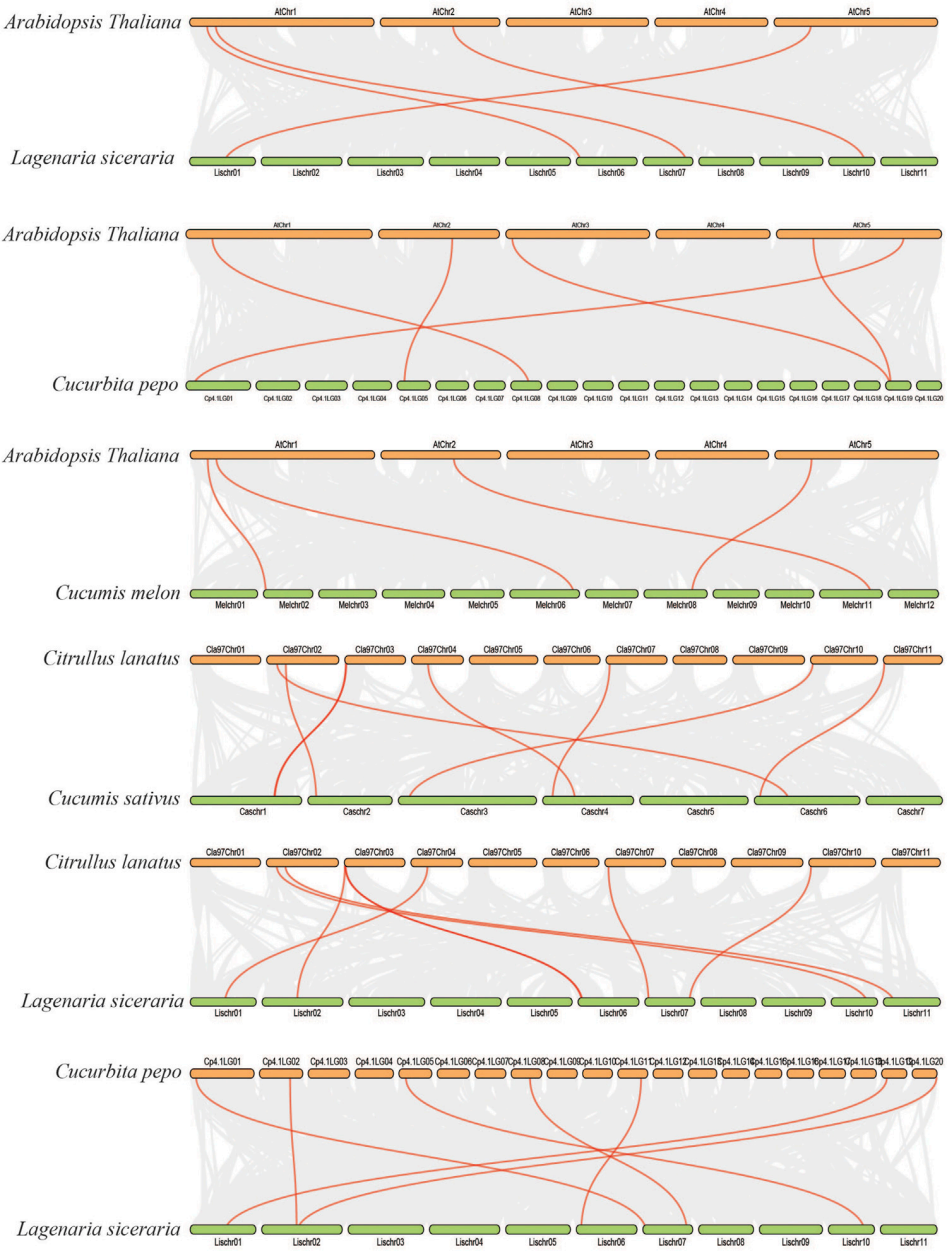
Collinearity relationships in five Cucurbitaceae genomes and *Arabidopsis thaliana*; different line color represents synteny relationships in the five Cucurbitaceae genomes.

We further analyzed the collinearity relationships of *SOD* genes between five Cucurbitaceae species (*Citrullus lanatus*, *Cucurbita pepo*, *Cucumis sativus*, *Lagenaria siceraria*, *Cucumis melo*) as these five plants belong to the Cucurbitaceae family and shared a similar antique (Supplementary Table S6) (Figure 7). A total of 35 collinear gene pairs were found between the five Cucurbitaceae genomes, including 5 orthologous gene pairs between *Arabidopsis* and *Cucurbita pepo*, 4 orthologous gene pairs between *Arabidopsis* and *Lagenaria siceraria*, 4 orthologous gene pairs between *Arabidopsis* and *Cucumis melo*, 8 orthologous gene pairs between *Citrullus lanatus* and *Cucumis sativus*, 7 orthologous gene pairs between *Citrullus lanatus* and *Lagenaria siceraria*, and 7 orthologous gene pairs between *Cucurbita pepo* and *Lagenaria siceraria*, suggesting a close relationship among the five Cucurbitaceae genomes. The results show that the genetic relationship between *SOD* gene pairs in *C. lanatus*, *C. pepo*, *C. sativus*, *C. melo*, and *L. siceraria* is close. No pairs of collinear *SODs* are shared between *Arabidopsis* and five Cucurbitaceae genomes, indicating the long distance phylogenetic relationship between two species.

### Gene Ontology Annotation Study of *SOD* Gene Family

**FIGURE 8:**
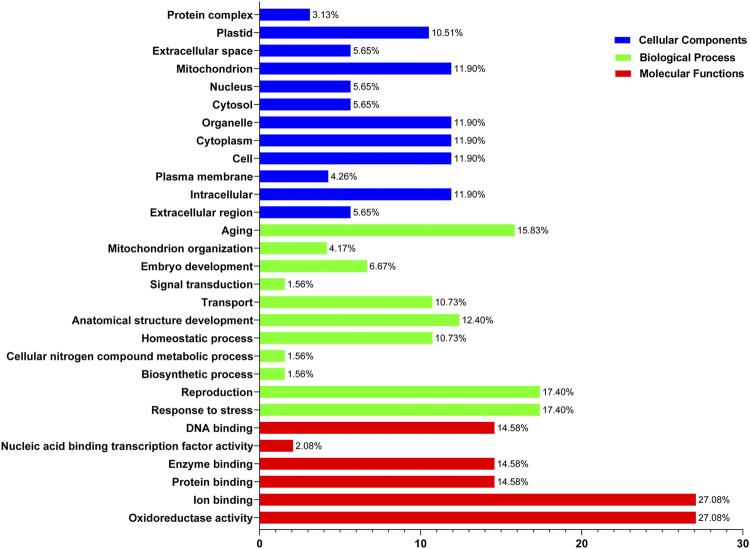
Gene Ontology (GO) annotation results of SOD genes of five species in the Cucurbitaceae family. GO analysis of SOD protein sequences is analyzed for their involvement or function in three important categories: biological process, molecular function, and cellular component.

Functional enrichment analysis of *SOD* genes was performed using DAVID. The *SOD* genes were categorized into three functional groups—biological processes, molecular functions, and cellular components—that are characteristics of genes or gene products, which enable us to understand the diverse molecular functions of proteins (Altschul et al., 1990). These results may be related to protein sequence similarities caused by genomic events (8, Supplementary Table S7). Evaluation of the biological processes mediated by *SODs* indicated that the same percentage (∼27%) of proteins was involved in oxidoreductase activity and ion binding. Among the SOD proteins, ∼14.58% of members showed potential involvement in protein binding, enzyme binding, and DNA binding, respectively, while nucleic acid binding transcription factor activity involvement is ∼2.08% during the 8 Lsi*SOD* genes of *Lagenaria siceraria* life cycle (Figure 8). At the same time, in *SOD* genes of five Cucurbitaceae species, “Cellular Components” includes 17.40% response to stress and reproduction, respectively. Meanwhile, 15.83% of genes were involved in aging, and 10.73% of genes were involved in homeostatic process and transport, respectively (Figure 8). Furthermore, the *SOD* genes of 8 Lsi*SOD* genes of *Lagenaria siceraria* were involved in the molecular functions, 11.9% of the genes were involved in intracellular, cell, cytoplasm, organelle, and mitochondrion while 10.51% of the genes were involved in plastid and 5.65% were involved in extracellular region, cytosol, nucleus, and extracellular space, respectively (Figure 8). The results corroborated the putative *SOD* promoter analysis (Jagadeeswaran et al., 2009; Jia et al., 2009; Guan et al., 2013; Manzoor et al., 2021a). Furthermore, analysis of the molecular function annotations revealed that all of the *LsiSOD* protein functions were enriched in SOD activity and metal ion binding.

### Genome-Wide Analysis of miRNA Targeting *LsiSOD* Genes

**FIGURE 9:**
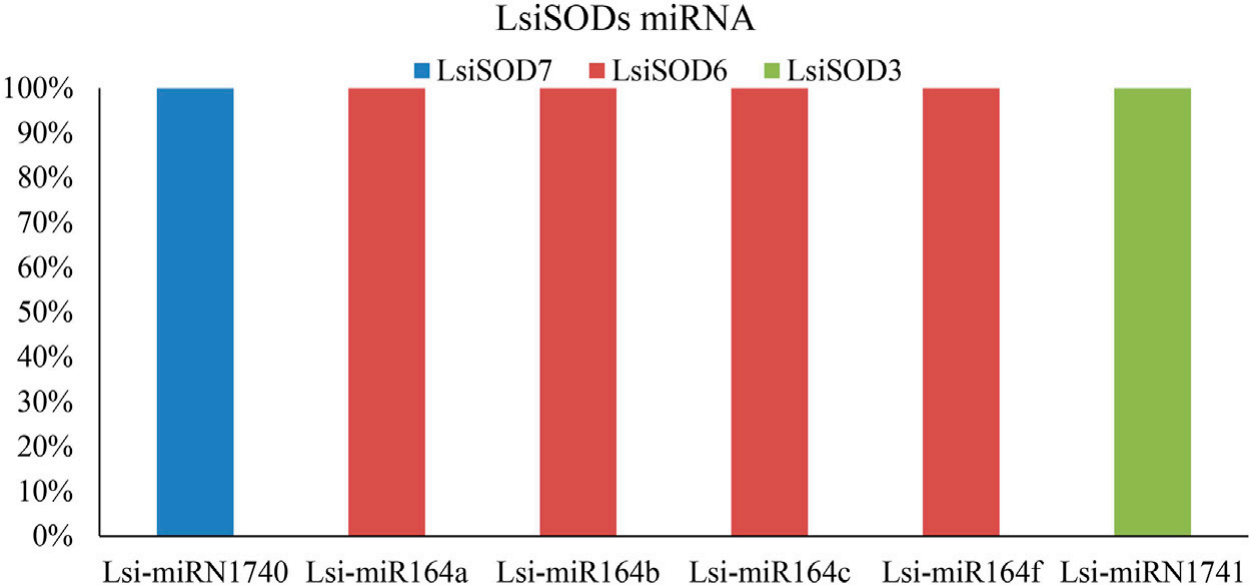
SODs targeted by miRNAs of *Lagenaria siceraria* (bottle gourd). The RNA sequence of each complementary site from 5′ to 3′ and the predicted miRNA sequence from 3′ to 5′ are shown in the expanded regions.

MicroRNAs (miRNAs) are endogenous microRNAs that play an important role in plant growth/development and stress responses. Thus, for a deep understanding of miRNA-mediated post-transcriptional regulation of *LsiSOD*, we identified 6 miRNAs (Lsi-MIR164a, Lsi-MIR164b, Lsi-MIR164c, Lsi-MIR164f, Lsi-miRN1740, and Lsi-miRN1741) with different classes of miRNA families (Figure 9; Supplementary Table S8). The analysis indicated that 3 *LsiSOD* genes (LsiSOD3, LsiSOD6, and LsiSOD7) were targeted on different miRNA. The expression profiles of these miRNAs and their targets are needed to detect and verify in further experiments to determine their biological functions in bottle gourd. The regulation of *SOD* genes’ expression by miRNA needs to be studied further.

### Tissue-Specific Expression of *LsiSOD* Genes

**FIGURE 10:**
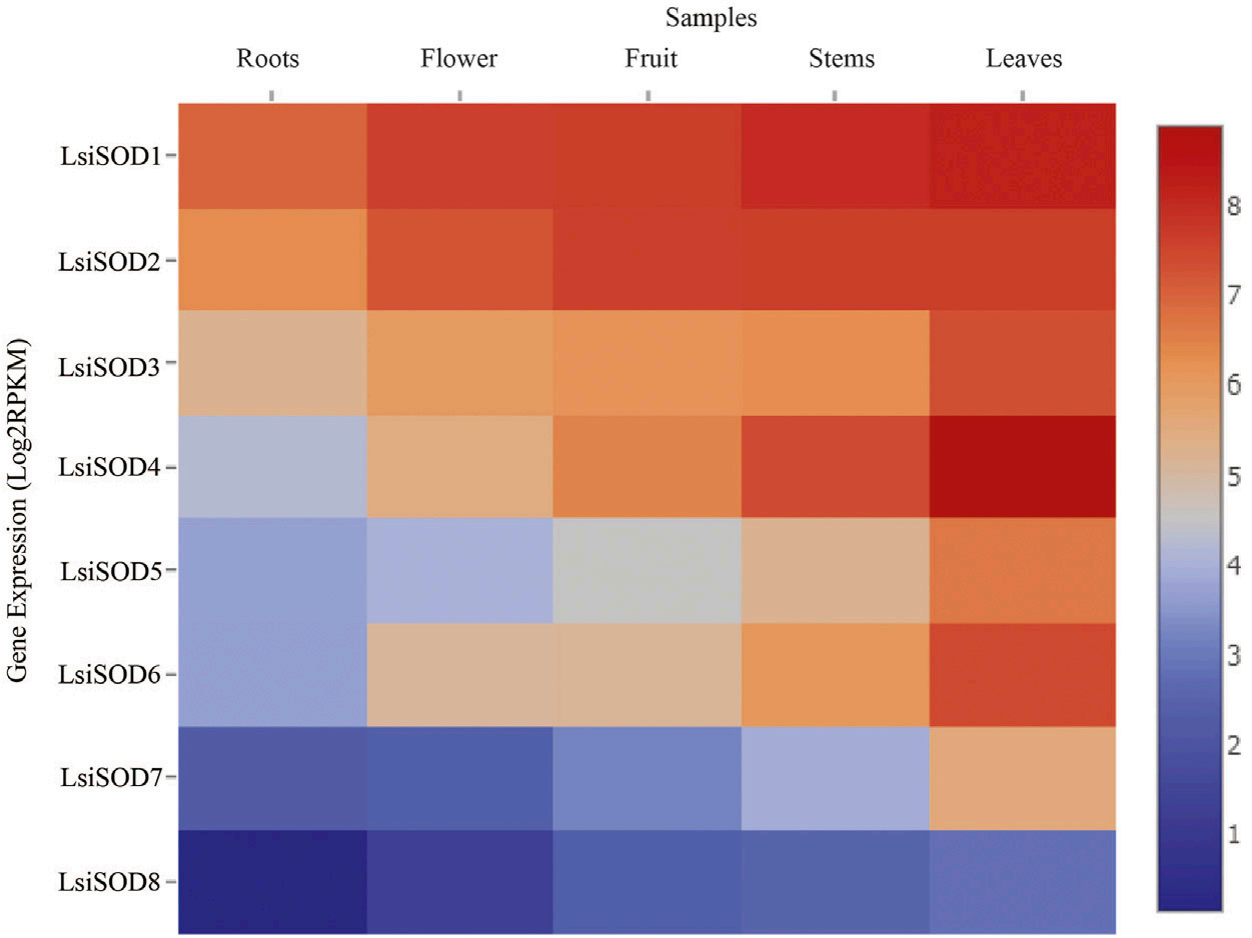
Relative expression profiles of SOD genes of five species in Cucurbitaceae family during different developmental stages. The FPKMs calculated by RNA-seq data are shown as a heat map. The color scale is shown at the side of the heat map reflecting the higher and lower relative abundance in each tissue.

A strong link between gene expression and function has been suggested and that the SOD gene family is primarily involved in plant growth, development, and stress responses. To determine the biological functions in *Lagenaria siceraria*, the expression profiles of the 8 *LsiSOD* genes were analyzed in tissues (root, flower, fruit, stem, and leaf) using RNA-seq data downloaded from CuGenDB (http://cucurbitgenetics.org/) (Zheng et al., 2019). These results indicated that *LsiSOD* genes have tissue-specific expression patterns. However, *LsiSOD*4, *LsiSOD*5, and *LsiSOD*6 exhibited low transcript abundance in root and flower while their highest expression was detected in stems and leaves. This suggested that they play an important role in the development of plants. In addition, *LsiSOD1*, *LsiSOD2*, and *LsiSOD3* are highly expressed in all parts of plants especially in the early stages of plant development (Figure 10). All *LsiSOD* genes were widely expressed and showed tissue-specific expression patterns while *LsiSOD8* shows less expression in all different developmental parts.

### Gene Expression Analysis of *LsiSOD* Gene Under Abiotic Stresses

**FIGURE 11:**
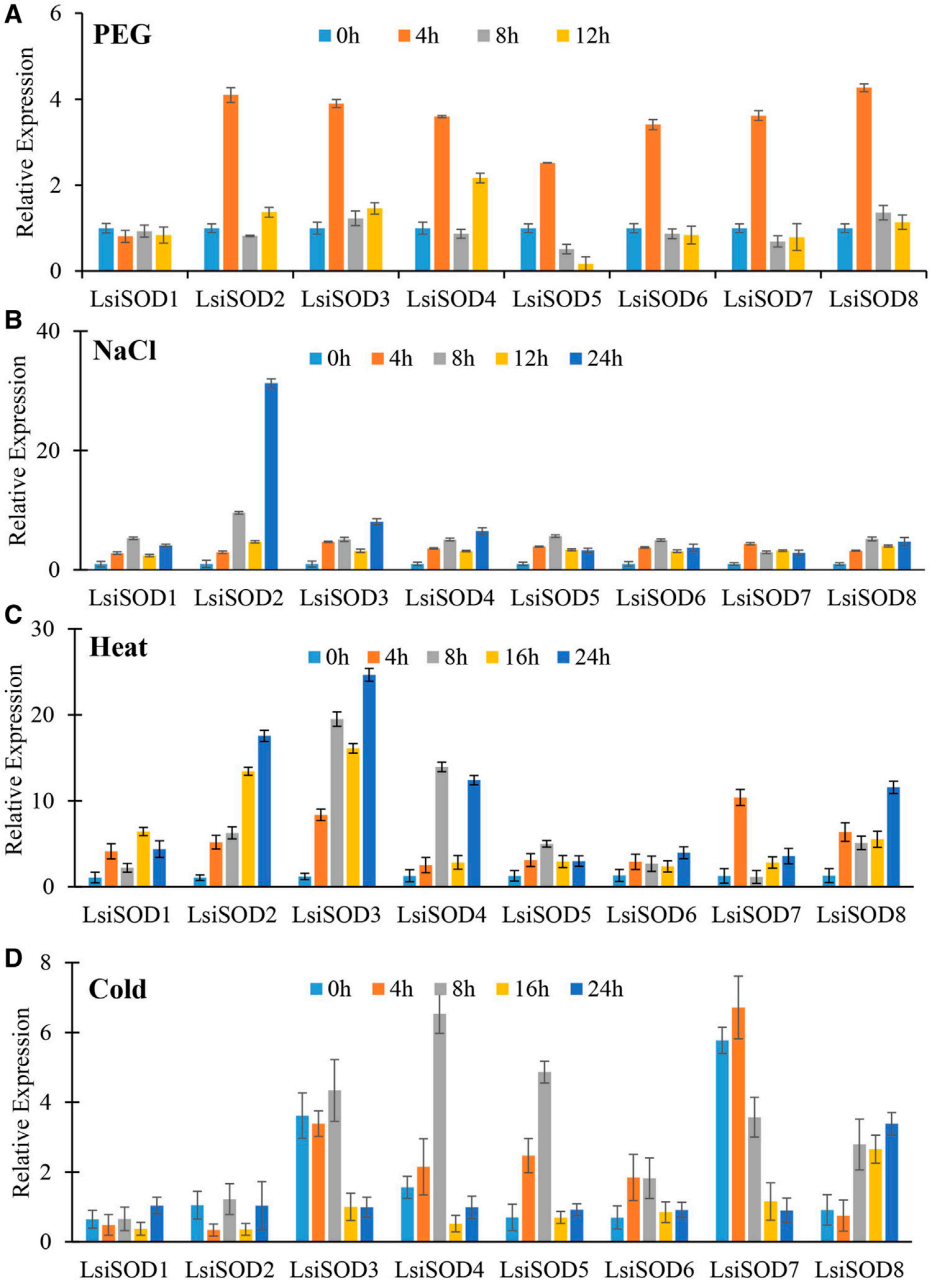
qRT-PCR study of SOD genes of bottle gourd in response to different abiotic stresses including **(A)** heat, **(B)** cold, **(C)** PEG, and **(D)** NaCl at different time points (0, 4, 8, 16, 24 h). Error bars indicate ±SD based on three biological replicates.

To understand the role of the *SODs*, qRT-PCR was used to analyze the expression patterns of the *SOD* gene in response to stress from heat, cold, drought, and NaCl. Significant differences were observed in the expression levels of the *LsiSOD* genes under different treatments, and complexity was detected in their expression patterns (Figure 11). The 8 *LsiSOD* genes were induced by heat treatment, and their expression profiles were significantly enhanced. Among them, at 4 h of treatment one gene *LsiSOD7* (2.62-fold), at 8 h of treatment two genes *LsiSOD3* (20.35-fold) and *LsiSOD4* (14.62-fold), at 16 h of treatment two genes *LsiSOD*2 (13.22-fold) and *LsiSOD*3 (16.41-fold), and at 24 h 2 genes *LsiSOD2* (18.24-fold) and *LsiSOD3* (24.16-fold) reached the highest expression level respectively. Interestingly, the transcription of *LsiSOD*3 and *LsiSOD*4 was gradually induced to 8 h, then decreased at 16 h, and finally reached a high level at 24 h, as described in Figure 11. Besides, in the expression of *LsiSOD*7, a dramatic increase was observed, which reached the maximum level at 4 h and decreased significantly at 8 h, then increased gradually with the treatment (Figure 11A). During cold treatment at 4 h, many *LsiSOD* genes were downregulated, *LsiSOD*3 (1.17-fold) remained unchanged, *LsiSOD*7 (1.27-fold) slightly increased, and then *LsiSOD*3 (7.27-fold) increased significantly at 8 h (Figure 11B). After 8 h, except for the increased expression of *LsiSOD*2 (1.71-fold) at 16 h, the other *LsiSOD* genes were significantly downregulated at 16 h and continued to decline at 24 h as compared with 0 h. After 8 h, the expression of *LsiSOD*7 (4.53-fold) and *LsiSOD*8 (4.39-fold) was progressively induced, and the induction peak appeared at 16 and 24 h, respectively. Under the PEG stress treatment, almost all *LsiSOD* gene expressions increased significantly at 4 h except *LsiSOD1*, and then the level of transcription decreased. At the same time, *LsiSOD1* decreased somewhat at 4 h, and its transcription remained unchanged (Figure 11C). At 4 h of treatment with PEG, the expression of *LsiSOD8* (4.27-fold) was maximum, whereas the expression of *LsiSOD8* (1.36-fold) was highest during 8 h of treatment, while at 12 h of treatment *LsiSOD4* (2.17-fold) shows maximum expression. During NaCl stress treatment, mostly the expression pattern of all *LsiSOD* genes increased tremendously at 8 h, decreased at 16 h, and finally increased at 24 h (Figure 11D). At 4 h of treatment, expression of *LsiSOD*3 increased (3.71-fold), and at 8 h of treatment with NaCl, expression of *LsiSOD2* increased (8.59-fold), while at 12 h of treatment, the expression of *LsiSOD*8 increased (3.01-fold) and the expression of *LsiSOD4* increased (4.53-fold). It should be worth mentioning that *LsiSOD*2 transcription level increased significantly in 24 h, which was significantly higher than other *LsiSOD* genes.

## Discussion

Environmental pressure poses a considerable challenge to crop production. Superoxide dismutase activity plays an important role in plant resistance to different stresses, such as salt, drought, and metal toxicity (Alscher et al., 2002; Schützendübel and Polle, 2002; Fernández-Ocaña et al., 2011; Atkinson et al., 2013; Lin and Lai, 2013; Wang et al., 2018). *SOD* gene family is widely distributed among many plant species, including *Arabidopsis* (Kliebenstein et al., 1998), rice (Dehury et al., 2013), longan (Lin and Lai, 2013), banana (Molina-Rueda et al., 2013), poplar (Gosavi et al., 2014), sorghum (Nath et al., 2014), cotton (Feng et al., 2015), and tomato (Filiz and Tombuloğlu, 2015; Zhang et al., 2016; Wang et al., 2016; Wang et al., 2017). Our study provides a systematic and comprehensive whole-genome evolutionary analysis of SOD members obtained from five Cucurbitaceae genomes while the focus of our study is on *Lagenaria siceraria*, which is one of the excellent vegetables that contain all the essential ingredients necessary for human health and quality of life (Division and Jambheshwar, 2011). *SODs* are identified as a crucial enzyme involved in many oxidation processes and protect the plant against ROS (Alscher et al., 2002). Therefore, we systematically analyzed the *SOD* gene family of Cucurbitaceae and determined the gene expression patterns in *L. siceraria* under different abiotic stresses (drought, heat, salt, and cold).

A phylogenetic study was conducted on SOD proteins in *Lagenaria siceraria* and four other cucurbit plants, namely, *Citrullus lanatus*, *Cucurbita pepo*, *Cucumis sativus*, and *Cucumis melo*. *SODs* could be classified into eight groups with good statistical support in line with previous studies (Alscher et al., 2002; Feng et al., 2015; Filiz and Tombuloğlu, 2015; Feng et al., 2016; Wang et al., 2017; Verma et al., 2019). *SOD* genes are localized to chloroplasts, and mitochondria were classified into the same subgroups. Furthermore, nearly all *LsiSOD*s are systematically grouped with at least one member of all plant species under consideration, highlighting the functional conservation of *LsiSOD*s with other plant species *SODs* (Figure 1).

In this study, *SOD* gene families were found in Cucurbitaceae genome, including *Citrullus lanatus*, *Cucurbita pepo*, *Cucumis sativus*, *Lagenaria siceraria,* and *Cucumis melo*. There are considerable differences in the size of genomes and the number of *SODs* in these plant organisms, but there is no substantial dependence on the size of genomes. The number of amino acids of SOD proteins ranged from 52 to 530 kDa, showing a significant variation. The difference in the number of *SOD* genes in different plant species may be due to gene duplication. In addition, the difference between clades might be related to different functions and diversity of exons/introns and conserved motif structure. Intron and exon variations play a major role in the evolution of different genes. The variation in introns/exons and motif structure of *SOD* genes suggests a high level of complexity between Cucurbitaceae species.

We further investigated the diversity between *SODs* by analyzing their subcellular localization as localization plays a vital role in their functions. The cytoplasm (34%), chloroplast (33%), mitochondria (22%), and nucleus (11%) *SOD* gene localizations were predicted in five Cucurbitaceae species (Supplementary Table S8), and further study is needed to confirm those localizations. Increasing evidence points to an important role of miRNAs in stress tolerance. Several studies have reported that miRNAs regulate the expression of stress-responsive protein-coding genes at post-transcriptional level, showing a reverse correlation between the miRNA and the target expression (Sunkar et al., 2007; Lomakina, 2018; Manzoor et al., 2020). These miRNAs resulted from computational predictions and deep sequencing, and they are involved in some biological processes reported in plants, including responses to environmental stresses and regulating cell growth, development, and metabolism (Qiu et al., 2007; Li et al., 2012). In the present study, we identified 3 miRNAs targeting 8 *LsiSOD* genes (Figure 9; Supplementary Table S8). In *Lagenaria siceraria*, the miR164 family comprises 3 members that generate four mature products, miR164a/b/c and miR164f, which could target at least three LsiSOD genes. The miR164 family is a highly conserved miRNA that has been found in many plant species. miRNA164a thereby increased the tolerance of the plant to the abiotic stress or increasing the biomass, vigor, or yield of the plant. The miRNA miR164 plays a central role during the development of serrated leaf margins in *Arabidopsis*. In this study, transcripts of three miRNA families were identified because miRNA expression is highly regulated under different abiotic stress conditions (Figure 9). Further studies are needed to determine the role of *SODs* in *Lagenaria siceraria.*

Gene duplications are an essential mechanism for creating genetic novelty in all plants, which could help organisms to adapt to environmental change (Alvarez-Buylla et al., 2000; Kondrashov, 2012; Manzoor et al., 2020). There are different kinds of gene duplication, including dispersed duplication (DSD), transposed duplication (TRD), and whole-genome duplication (WGD), which differentially contribute to the expansion of plant-specific genes (Qiao et al., 2018, Qiao et al., 2019). Gene replication can cause variation in the number of *SOD* genes in different plant species, and involves tandem and segmented replication, which plays an important role in *SOD* gene diversity, expansion, and duplication (Filiz and Tombuloğlu, 2015; Wang et al., 2016; Zhang et al., 2016; Wang et al., 2017). Thus, tandem repetition is likely to play a vital function in the amplification of the *LsiSOD*s, for example, the two neighboring genes *LsiSOD*3 and *LsiSOD*6 on Chr6 (Figure 3). Characterization of the gene structure describes that the number of introns from the *SOD* gene family differed between the observed Cucurbitaceae species (Figure 2); in addition, the number of introns in *L. siceraria* was 6–8. Previous studies have shown that the plant’s *SOD* gene has a strongly conserved intron pattern and that seven introns are present in most cytoplasmic and chloroplast SODs (Fink and Scandalios, 2002). In our study, only the *LsiSOD* members were predicted to contain seven introns, as shown in Figure 2.

Analysis of the *cis*-elements in SOD gene promoters resulted in the detection of three major types of *cis*-elements associated with light, abiotic stress, and hormone response as well as *cis*-elements related to developmental processes and tissue-specific expression. A relatively large number of light-responsive *cis*-elements were detected in *SOD* gene promoters, suggesting that SODs might participate in the abiotic response. Some studies have shown that SOD genes are involved in the response to abiotic stress in different plants like *Dendrobium catenatum*, *Pennisetum glaucum*, maize, and *Arabidopsis* (Divya et al., 2019; Huang et al., 2020; Kliebenstein et al., 2020; Liu et al., 2021). In addition, a series of *cis*-elements related to abiotic stress responses were identified in SOD gene promoters, such as MBS, ERE, TC-rich repeats, ARE, ABRE, and Box-4, which may regulate gene expression under various stresses. Most of the SOD genes in *Arabidopsis*, banana, rice, tomato, poplar, cotton, and other different plants can be induced in response to various abiotic stresses such as heat, cold, drought, and salinity (Kliebenstein et al., 1998; Lee et al., 2007; Dehury et al., 2013; Nath et al., 2014; Feng et al., 2016; Wang et al., 2016; Zhang et al., 2016; Wang et al., 2017; Verma et al., 2019). GO annotation analysis further verified these results (Supplementary Table S7). In addition, some researchers have reported similar findings in different crops and found that *SOD* gene plays an important role under different stress conditions. GO annotation results confirmed the *LsiSOD*’s role in response to different stress stimuli, cellular oxidant detoxification processes, metal ion binding activities, SOD activity, and different cellular components. These results can promote our understanding of *LsiSOD* genes under different environmental conditions. We also found protein similarities (Supplementary Table S9) by using protein–protein interactions (PPIs) handle a wide range of biological processes, including metabolic and developmental control and cell-to-cell interactions (Mittler, 2017). Regarding the possible role of *SODs* from Cucurbitaceae, the expression profile of *SODs* in different tissues was analyzed based on sequence evidence from RNA-seq data. Data analysis suggests that the gene family of *SOD* was expressed in all tissues; some *SODs* had tissue-specific expression patterns (Figure 10).

### Expression Patterns of *SOD* Gene Family of *Lagenaria siceraria* Under Heat, Cold, Drought, and Salt Stress

Excess ROS resulting from abiotic stress can pose threats to *L. siceraria* yield. The SODs play an active role in ROS removal from plants caused by various abiotic stresses (Gill and Tuteja, 2010). However, *LsiSOD*s’ specific response to heat, cold, drought, and salt is not well understood. Therefore, the analyses of qRT-PCR provide important clues for understanding the possible role of *LsiSOD*s under various stresses. Based on the evaluation of *cis*-elements of *LsiSOD* gene promoter, three main *cis*-element types related to light, abiotic stress, and hormone response, as well as *cis*-elements related to development process, were determined. The *cis-*elements in *LsiSODs* promoter include TC-rich motif, LTR motif, MBS motif, ARE motif, and ABRE motif, which is the evidence of abiotic stress response (Supplementary Table S4). These motifs were previously observed in different plant species to cope with abiotic stresses, such as bananas, tomatoes, Brassica, tobacco, and millet (Oppenheim et al., 1996; Kurepa et al., 1997; Kliebenstein et al., 1998; Pilon et al., 2011; Feng et al., 2015; Wang et al., 2018; Hu et al., 2019; Verma et al., 2019). In this study, the expression level of eight *LsiSOD* genes also changed significantly under different stresses, indicating that these genes play an important regulatory role in stress response and may have a certain functional relationship. Almost all *LsiSOD* genes were upregulated during heat treatment, and some displays have similar expression (Figure 11C).

Under cold stress, the expression level of all *LsiSOD* genes is unregulated significantly, and their patterns of expression are different from each other, as shown in Figure 11D, which means that *LsiSOD* genes under cold stress may have functional diversity. Similar expression patterns were observed in *LsiSOD1* with *MaCSD1B* and *MaCSD2B* (Feng et al., 2015). In PEG treatment, almost all *Lsi*-*SOD1* genes have identical expression patterns, reach the highest level at 4 h, and then decline, as shown in Figure 11A, meaning that the function of the *LsiSOD* gene is related to drought.

The expression of most of the *LsiSOD* genes changed during NaCl treatment; *LsiSOD*2 is the only one of the eight *LsiSOD* genes with a significant increase, as shown in Figure 11B, indicating that *LsiSOD*2 plays an active role in detoxification of ROS during salt stress. Similar results were observed during salt stress in tomato (Feng et al., 2016). *SlSOD1* and *LsiSOD*2 were grouped in the same subgroup, showing approximately high amino acid sequence homology. Altogether, we conclude that the *LsiSOD* gene plays a specific role in ROS removal induced by abiotic stress, which enhances plant adaptability to stress. In addition, some *LsiSOD* genes were correlated to abiotic stress exhibiting different expression patterns. For example, cold treatment reduced the expression of *LsiSOD1*, heat treatment and NaCl treatment increased the expression of *LsiSOD1*, while under PEG stress, the expression level remained the same without significant change, as shown in Figure 11, which means that the function of *LsiSOD1* in different signaling pathways was different. It needs to be further explored to elucidate the role of the *LsiSOD* gene in *L. siceraria* under various abiotic stresses.

## Conclusion

In this study, we identified *SOD* genes from the Cucurbitaceae family and analyzed their genomic structure, GO annotation analysis (molecular processes, biological functions, and cellular components), miRNA, gene duplication events (TD, PD, DSD, WGD, and TD), conserved motif patterns, phylogenetic relationships, mode of gene duplications, subcellular localization, and RNA-seq data analysis. qRT-PCR was used to evaluate the *LsiSOD* gene regulatory response under a variety of abiotic stresses, such as heat, cold, PEG, and NaCl. This research provided insight and further functional identification of the Cucurbitaceae family of *SOD* genes and laid the framework for understanding the molecular mechanism of the *SOD* gene in response to stress and plant growth. Genome-wide study of *SOD* genes provides insights into the evolutionary history and has laid a foundation for gene role, functional characteristics, and molecular mechanism in the plant development process and stress response (Mistry et al., 2021).

## Data Availability

The datasets presented in this study can be found in online repositories. The names of the repository/repositories and accession number(s) can be found below: CuGenDB, PRJNA387615.
