# Supplementary material for: Genome-Wide Evolution and Comparative Analysis of Superoxide Dismutase Gene Family in Cucurbitaceae and Expression Analysis of Lagenaria siceraria Under Multiple Abiotic Stresses
Source: Front Genet. 2022 Feb 8;12:784878. doi: 10.3389/fgene.2021.784878 (PMC8861505; doi:10.3389/fgene.2021.784878)
Supplement: Supplementary file 2 [file Table1.DOCX]

| **Primer name** | **Sequence (5’-3’) F** | **Function** |
| --- | --- | --- |
| Lsi01G016730 | F ATGCCTCCAAACAAAACCATT  R GGACTCCGACGAACTTGTTG | qRT-PCR |
| Lsi11G004820 | F AGCAATCAGGGAGTCAGTGG  R CCAAGATCACCAACATGACG | qRT-PCR |
| Lsi06G001840 | F CCAAGAAGGAGATGGTCCAA  R CCATCTTCACCAGCGGTAAT | qRT-PCR |
| Lsi06G001600 | F CAACCTGGAGGTGGAAACAT  R CCAAAGAAGTGGGCTTATCG | qRT-PCR |
| Lsi10G012210 | F GAGGATGAAATCCGTCATGC  R GTTGCCAGTGGTTGAACTGAG | qRT-PCR |
| Lsi07G013670 | F TGACTGAAGCTTTGGAGCAGAC  R GATTGACCAACCATGTTTTCCA | qRT-PCR |
| Lsi02G012110 | F CGATTCCCAGTTCGGTTCTC  R TGCATGCTCCCAAACATCTATC | qRT-PCR |
| Lsi07G001390 | F CATCTATCGCTTTGCCACCT  R GTTCCAAAGCATCCAGAGGA | qRT-PCR |
| Lsi-Actin | F GAATCCAGCACGATACCA  R TCAACCCAAAGGCTAACA | qRT-PCR |

**Table S1**. List of the primers used in gene expression analysis.
