# Supplementary figures and images for: Genome-Wide Evolution and Comparative Analysis of Superoxide Dismutase Gene Family in Cucurbitaceae and Expression Analysis of Lagenaria siceraria Under Multiple Abiotic Stresses
Source: Front Genet. 2022 Feb 8;12:784878. doi: 10.3389/fgene.2021.784878 (PMC8861505; doi:10.3389/fgene.2021.784878)

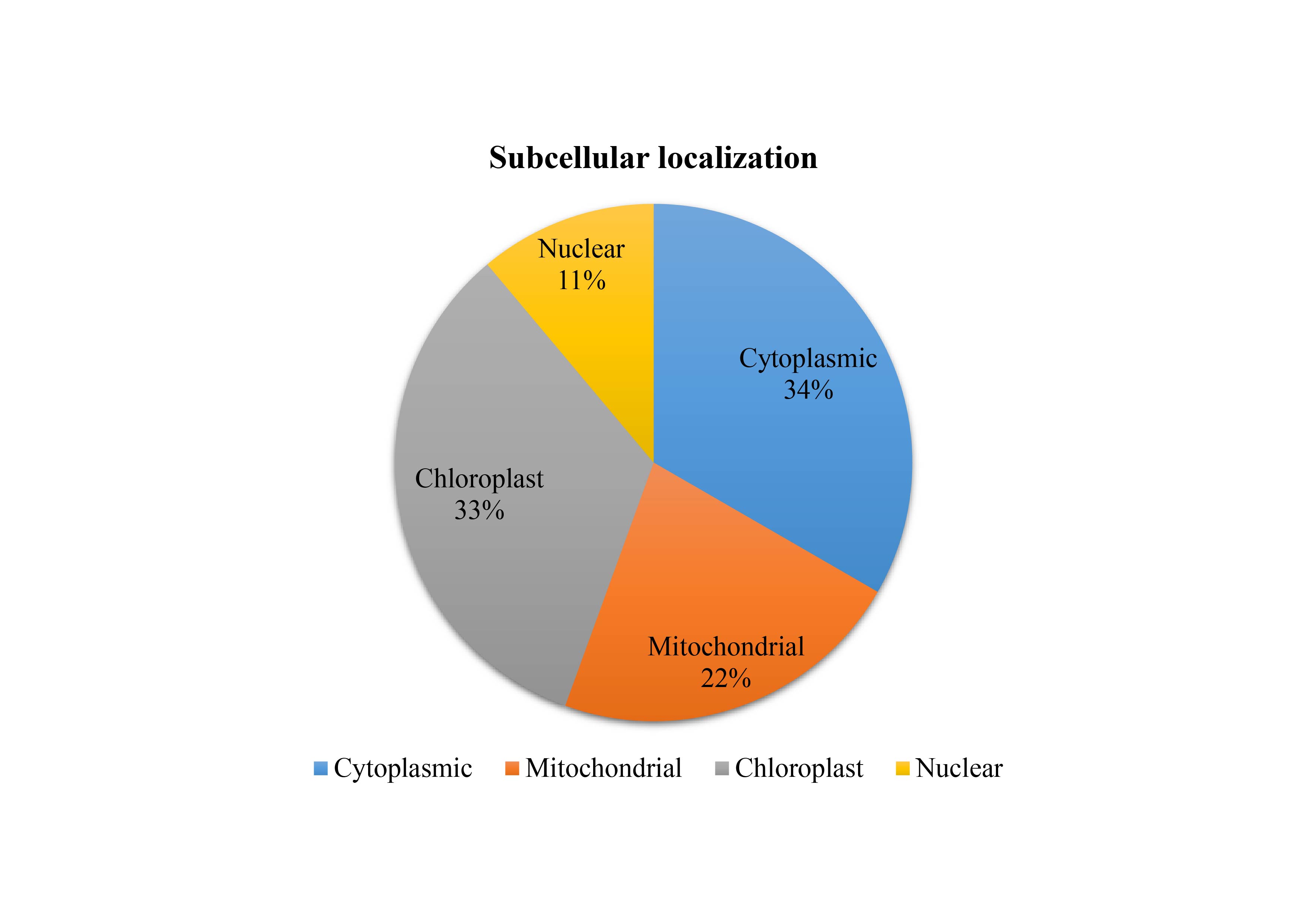

Supplement: Supplementary file 4 [file Image1.JPEG]

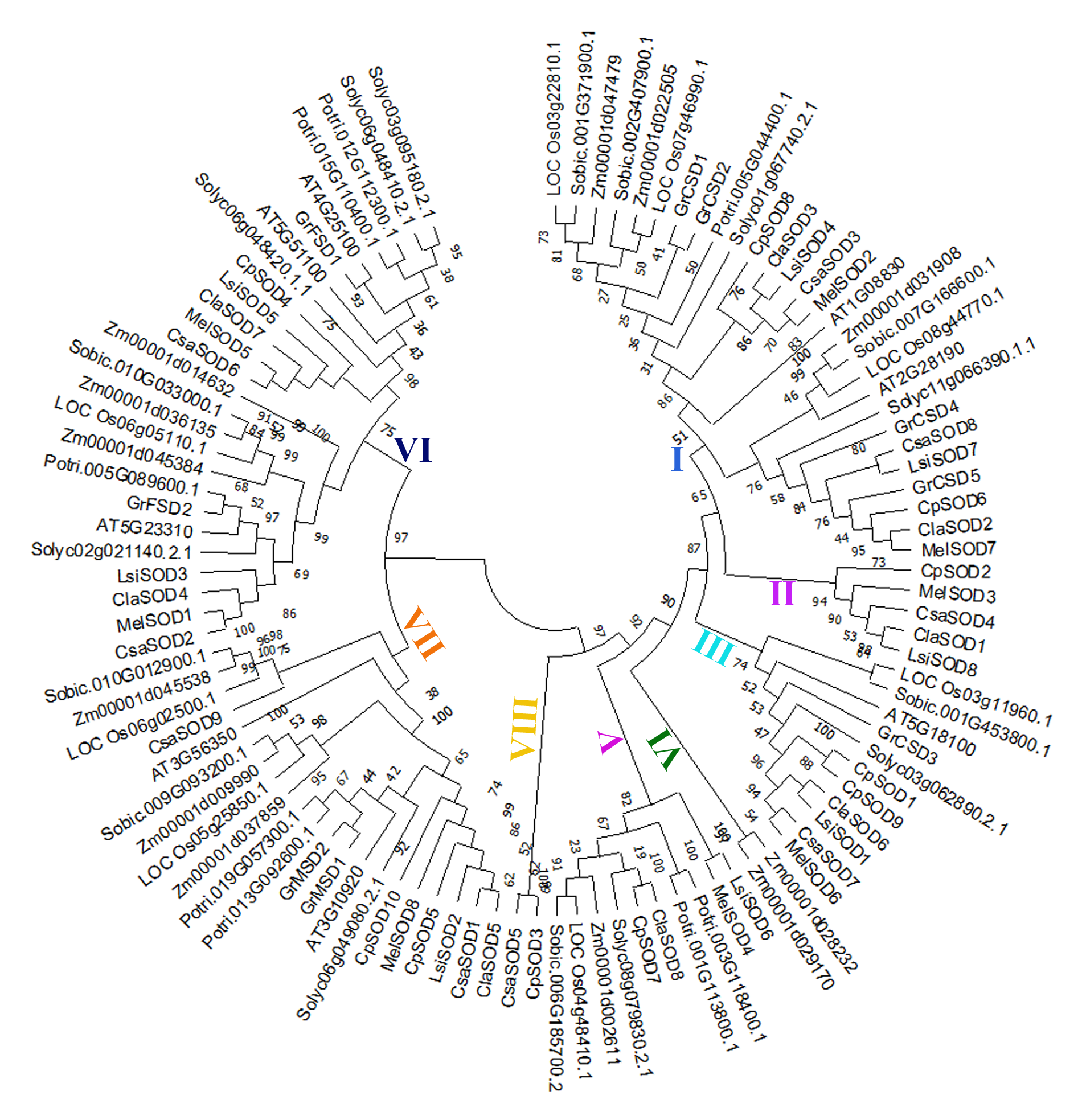

Supplement: Supplementary file 5 [file Image2.TIF]
