# Supplementary material for: Genome-Wide Evolution and Comparative Analysis of Superoxide Dismutase Gene Family in Cucurbitaceae and Expression Analysis of Lagenaria siceraria Under Multiple Abiotic Stresses
Source: Front Genet. 2022 Feb 8;12:784878. doi: 10.3389/fgene.2021.784878 (PMC8861505; doi:10.3389/fgene.2021.784878)
Supplement: Supplementary file 7 [file Table2.DOCX]

|  | **Watermelon^1^** | **Zucchini^2^** | **Cucumber^3^** | **Bottle gourd^4^** | **Melon^5^** |
| --- | --- | --- | --- | --- | --- |
| 1 | Cla97C02G042100  ClaSOD1 [Cu-Zn] | Cp4.1LG00g08890  CpSOD1 [Cu-Zn] | CsaV3_1G004740  CsaSOD1[Fe/Mn] | Lsi01G016730  LsiSOD1 [Cu-Zn] | MELO3C015351  MelSOD1 [Fe/Mn] |
| 2 | Cla97C02G047680  ClaSOD2 [Cu-Zn]/ | Cp4.1LG00g09240  CpSOD2 [Cu-Zn] | CsaV3_1G039270  CsaSOD2[Fe/Mn] | Lsi02G012110  LsiSOD2 [Mn] | MELO3C015374  MelSOD2 [Cu-Zn] |
| 3 | Cla97C03G066780  ClaSOD3 [Cu-Zn]/ | Cp4.1LG00g14260  CpSOD3 [Cu-Zn] | CsaV3_1G039510  CsaSOD3 [Cu-Zn] | Lsi06G001600  LsiSOD3 [Fe/Mn] | MELO3C004342  MelSOD3 [Cu-Zn] |
| 4 | Cla97C03G067030  ClaSOD4 [Fe/Mn] | Cp4.1LG01g03240  CpSOD4 [Fe/Mn] | CsaV3_2G004490  CsaSOD4 [Cu-Zn] | Lsi06G001840  LsiSOD4 [Cu-Zn] | MELO3C014007  MelSOD4 [Fe/Mn] |
| 5 | Cla97C03G050910  ClaSOD5 [Fe/Mn] | Cp4.1LG02g11270  CpSOD5 [Fe/Mn] | CsaV3_3G003410  CsaSOD5[Cu-Zn] | Lsi07G001390  LsiSOD5 [Fe/Mn] | MELO3C017624  MelSOD5 [Fe/Mn] |
| 6 | Cla97C04G071940  ClaSOD6 [Cu-Zn] | Cp4.1LG05g02890  CpSOD6 [Cu-Zn] | CsaV3_4G004420  CsaSOD6[Fe/Mn] | Lsi07G013670  LsiSOD6 [Cu-Zn] | MELO3C008809  MelSOD6 [Cu-Zn] |
| 7 | Cla97C07G144240  ClaSOD7 [Fe/Mn] | Cp4.1LG08g07900  CpSOD7 [Cu-Zn] | CsaV3_4G013220  CsaSOD7 [Cu-Zn] | Lsi10G012210  LsiSOD7 [Cu-Zn] | MELO3C026955  MelSOD7 [Cu-Zn] |
| 8 | Cla97C10G187890  ClaSOD8 [Cu-Zn] | Cp4.1LG11g09200  CpSOD8 [Cu-Zn] | CsaV3_6G014400  CsaSOD8 [Cu-Zn] | Lsi11G004820  LsiSOD8 [Cu-Zn] | MELO3C020487  MelSOD8 [Fe/Mn] |
| 9 |  | Cp4.1LG19g02010  CpSOD9 [Cu-Zn] | CsaV3_6G002070  CsaSOD9 [Cu-Zn] |  |  |
| 10 |  | Cp4.1LG20g07540  CpSOD10 [Fe/Mn] |  |  |  |

Table S2. List of SOD genes in five Cucurbitaceae species

Watermelon: Cla; *Citrullus lanatus*^1^, Zucchini: Cp; *Cucurbita pepo*^2^, Cucumber: Csa; *Cucumis sativus*^3^, Bottle gourd: Lis; *Lagenaria siceraria*^4^, Melon: Mel; *Cucumis melon*^5^.
